# Effectiveness of the standard and an alternative set of *Streptococcus pneumoniae* multi locus sequence typing primers

**DOI:** 10.1186/1471-2180-14-143

**Published:** 2014-06-03

**Authors:** Paul Adamiak, Otto G Vanderkooi, James D Kellner, Anthony B Schryvers, Julie A Bettinger, Joenel Alcantara

**Affiliations:** 1 Department of Microbiology, Immunology and Infectious Diseases, 3330 Hospital Dr. NW, Calgary, AB T2N 4 N1, Canada; 2 Alberta Children’s Hospital Research Institute for Child and Maternal Health, 3330 Hospital Dr. NW, T2N 4N1 Calgary, AB, Canada; 3 Department of Pediatrics University of Calgary, Alberta Children’s Hospital, 2888 Shaganappi Trail NW, T3B 6A8 Calgary, AB, Canada; 4 Department of Pathology and Laboratory Medicine Calgary Laboratory Services, 3535 Research Rd NW, T2L 2 K8 Calgary, AB, Canada; 5 Vaccine Evaluation Center BC Children’s Hospital, University of British Columbia, 4480 Oak St, V6H 3 V4 Vancouver, British Colombia, Canada

**Keywords:** Multi-locus sequence typing, MLST, Invasive pneumococcal disease, Molecular epidemiology, *Streptococcus pneumoniae*, Bacterial typing

## Abstract

**Background:**

Multi-locus sequence typing (MLST) is a portable, broadly applicable method for classifying bacterial isolates at an intra-species level. This methodology provides clinical and scientific investigators with a standardized means of monitoring evolution within bacterial populations. MLST uses the DNA sequences from a set of genes such that each unique combination of sequences defines an isolate’s sequence type. In order to reliably determine the sequence of a typing gene, matching sequence reads for both strands of the gene must be obtained. This study assesses the ability of both the standard, and an alternative set of, *Streptococcus pneumoniae* MLST primers to completely sequence, in both directions, the required typing alleles.

**Results:**

The results demonstrated that for five (*aroE*, *recP*, *spi*, *xpt*, *ddl*) of the seven *S. pneumoniae* typing alleles, the standard primers were unable to obtain the complete forward and reverse sequences. This is due to the standard primers annealing too closely to the target regions, and current sequencing technology failing to sequence the bases that are too close to the primer. The alternative primer set described here, which includes a combination of primers proposed by the CDC and several designed as part of this study, addresses this limitation by annealing to highly conserved segments further from the target region. This primer set was subsequently employed to sequence type 105 *S. pneumoniae* isolates collected by the Canadian Immunization Monitoring Program ACTive (IMPACT) over a period of 18 years.

**Conclusions:**

The inability of several of the standard *S. pneumoniae* MLST primers to fully sequence the required region was consistently observed and is the result of a shift in sequencing technology occurring after the original primers were designed. The results presented here introduce clear documentation describing this phenomenon into the literature, and provide additional guidance, through the introduction of a widely validated set of alternative primers, to research groups seeking to undertake *S. pneumoniae* MLST based studies.

## Background

Accurate, reproducible isolate characterization data helps epidemiologists, scientists, physicians, public health officials, and many other professions, better monitor and manage endemic and epidemic infectious disease trends [1]. Historically, bacterial typing schemes have been based on immunological and electrophoretic approaches [2]. Immunological based schemes classify strains on the specificity of antibodies raised against antigenic bacterial components. This approach has been widely applied in the form of capsular serotyping, whereby the antigenic specificity of different intra-species capsule types are used to classify the bacteria [3,4].

However, many globally significant bacterial pathogens such as *Streptococcus pneumoniae* and *Neisseria meningitidis* are readily able to incorporate environmental genetic material into their genomes allowing for rapid genetic variation and interchange of immunogenic components; including those on which serotyping is based [5]. This phenomenon has been observed recently with *S. pneumoniae* capsular typing following the introduction of the seven-valent pneumococcal conjugate vaccine (PCV7) [6]. As a result of the component specificity of immunological based typing methods, it has become well recognized that strains possessing the same serotype are not necessarily clonally related, nor expected to possess the same repertoire of virulence factors. Immunogenic approaches are now used in more focused ways to explore specific factors, particularly those relevant to guiding vaccine evaluation and development, as was demonstrated with a recent serotype B meningococcal vaccine investigation [7].

Multi-locus enzyme electrophoresis (MLEE) is another typing method, and is based on the relative electrophoretic mobility of a set of ubiquitously present bacterial enzymes [8]. This approach is not dependent on a single immunogenic component and as such is less influenced by horizontal exchange or positive selection events. However, it is complicated to perform and it is difficult to compare the resulting electrophoretic types between different groups [2]. Similar to the MLEE, pulse field gel electrophoresis (PFGE) classifies individual strains based on the gel electrophoretic mobility of bacterial components: in this case the relative mobility of DNA fragments which have been obtained through restriction enzyme digestion [9]. PFGE has been widely used for typing and has been considered a gold standard for some epidemiological studies, however, there have been challenges in standardizing protocols between different research groups [10].

Multi-locus sequence typing (MLST) is a classification scheme whereby isolates are typed based on the nucleotide sequences from a set of housekeeping genes that are necessary for the maintenance of basic cellular functions. The nucleotide sequences for each of these housekeeping genes are used to define a unique bacterial sequence type [1]. Each gene is sequenced from individual strains and then compared against existing sequences in a publically accessible, globally maintained database. Those submitted sequences matching ones already in the database are assigned the gene type number of the sequence in the database; if a novel sequence is submitted, the curator of the database assesses the sequencing results and assigns an appropriate gene number. While this approach does address several of the limitations encountered by other typing methods, the cost of sequencing can be a barrier to large scale typing projects. Particularly, because of the potential for error in sequencing reads the standard for determining a gene type requires matching forward and reverse sequences. The *S. pneumoniae* typing system is based on the partial sequence of seven genes coding for the housekeeping proteins: Shikimate dehyrogenase (*aroE*), glucose-6-phosphate dehydrogenase (*gdh*), glucose kinase (*gki*), transketolase (*recP*), signal peptidase I (*spi)*, xanthine phosphoribosyltransferase (*xpt*), and D-alanine-D-alanine ligase (*ddl*) [11].

Some preliminary results, and information provided by the curator of the *S. pneumoniae* MLST database indicated that several of the provided MLST sequencing primers were unable to obtain the full sequence required in each direction. As a result, in cases where a novel gene type is identified based on sequences from the standard primers (Table 1), the investigators are required to design new primers and re-sequence the particular gene (Cynthia Bishop, personal communication, May, 2012). In these circumstances, investigators are required to expend additional time and resources developing new primers, as well as purchasing additional sequencing and validating results. While several investigators in the field are aware of this issue, and all sequences in the MLST database have been correctly verified through subsequent primer redesign and re-sequencing, this limitation has not been specifically addressed in the literature [12,13] (Cynthia Bishop, personal communication, May 2012).

**Table 1:** **Standard *S. pneumoniae *** **amplification and sequencing primers proposed by Enright and Spratt **[11]

**Typing gene**	**Primer sequences**	**Annealing temperature °C**
*aroE* shikimate dehydrogenase	F: 5′-GCCTTTGAGGCGACAGC	55
	R: 5′-TGCAGTTCA(G/A)AAACAT(A/T)TTCTAA	
*gdh* glucose-6-phosphate dehydrogenase	F: 5′-ATGGACAAACCAGC(G/A/T/C)AG(C/T)TT	55
	R: 5′-GCTTGAGGTCCCAT(G/A)CT(G/A/T/C)CC	
*gki* glucose kinase	F: 5′-GGCATTGGAATGGGATCACC	60
	R: 5′-TCTCCCGCAGCTGACAC	
*recP* transketolase	F: 5′-GCCAACTCAGGTCATCCAGG	65
	R: 5′-TGCAACCGTAGCATTGTAAC	
*spi* signal peptidase I	F: 5′-TTATTCCTCCTGATTCTGTC	50
	R: 5′-GTGATTGGCCAGAAGCGGAA	
*xpt* xanthine phosphoribosyltransferase	F: 5′-TTATTAGAAGAGCGCATCCT	55
	R: 5′-AGATCTGCCTCCTTAAATAC	
*ddl* D-alanine-D-alanine ligase	F: 5′-TGC(C/T)CAAGTTCCTTATGTGG	65
	R: 5′-CACTGGGT(G/A)AAACC(A/T)GGCAT	

The lack of description of this limitation in the literature is evidenced by the prevalence of recent studies only referencing the original primers, and not providing any discussion pertaining to the sequencing challenge [6,14-18]. The purpose of this study is to systematically identify the primers unable to obtain the correct sequence, describe an alternative set of primers, and introduce documentation to the literature offering additional guidance to groups undertaking *S. pneumoniae* MLST studies. In this investigation, the effectiveness of the standard MLST sequencing primers, and an alternate set of primers were evaluated for their ability to completely sequence, in both directions, the appropriate typing regions of each gene.

## Results

**Figure 1:**
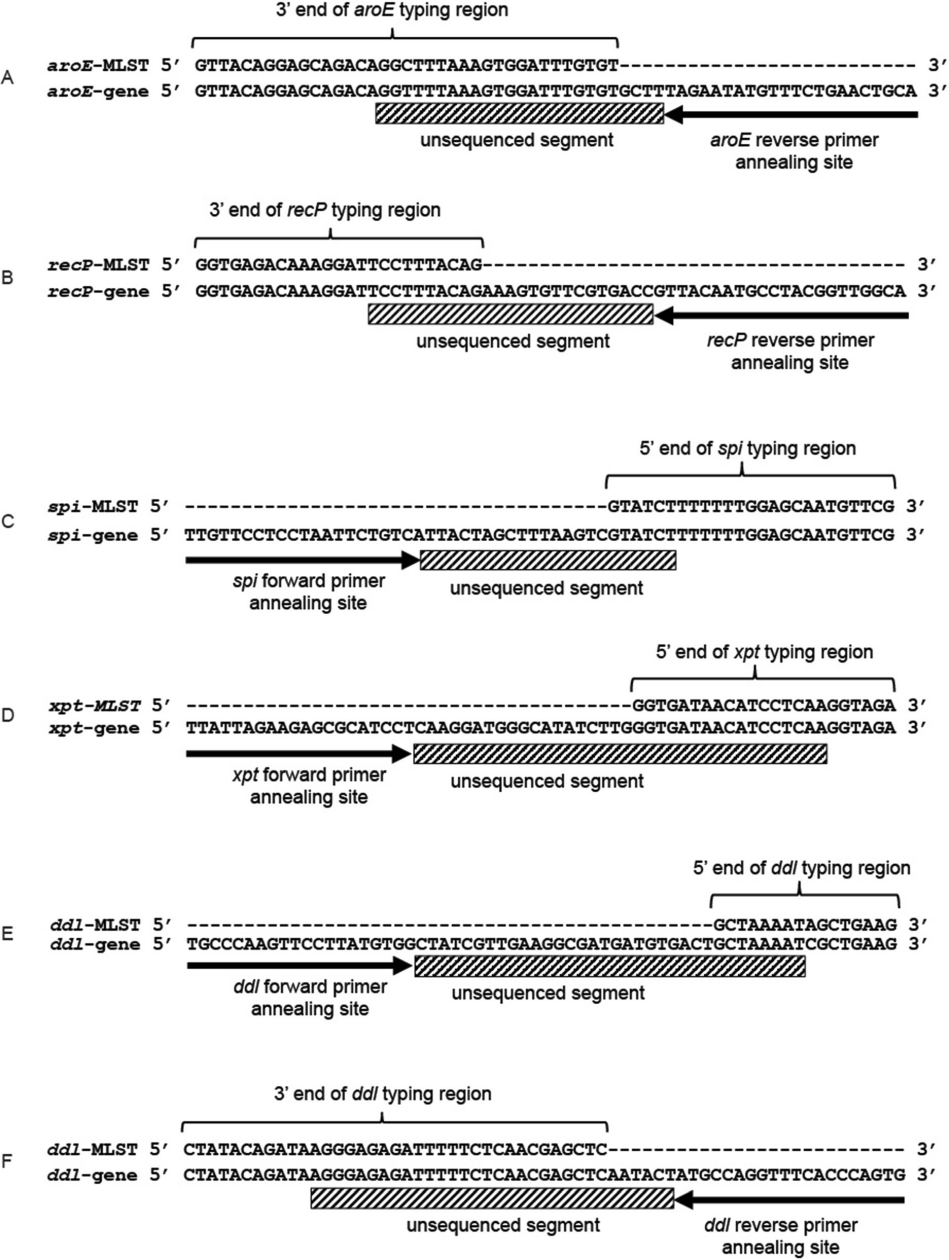
***S. pneumoniae *MLST typing regions for each of the segments not fully sequenced by the standard primers aligned with a section of the corresponding genomic DNA. **Panels **(A)** through **(F)** identify each individual gene and direction combination, for which the complete typing region is not obtained. The black arrows depict the binding sites of the standard primers to the up or downstream genomic DNA. The line marked boxes identify the segment that is consistently not obtained by sequencing with the standard primers. The angle bracket and top sequence identify either the 5’ or 3’ end of the typing region depending on the specific MLST gene.

This analysis consistently observed that the forward and reverse sequences obtained with the standard MLST primers only completely covered the typing region for two of the seven genes: *gki* and *gdh*. The reverse primer for the *aroE*, and *recP* genes failed to sequence the last 21 and 10 bases of their respective typing regions (Figure 1A, and B). The forward *spi* and *xpt* MLST primers do not sequence the first 6 and 17 bases of their respective typing regions (Figure 1C and D). In the case of *ddl*, the forward primer was unable to sequence the first 8 bases (Figure 1E) and the reverse did not sequence the last 26 bases (Figure 1F). These observations were consistent across all of the different isolates, both sequencing services, and each replicate. In each of the cases that the full sequence was not obtained, the alignment of the primers with publically available genomic sequences for *S. pneumoniae* identified that those primers annealed less than 30 base pairs from the required typing region (Figure 1).

**Figure 2:**
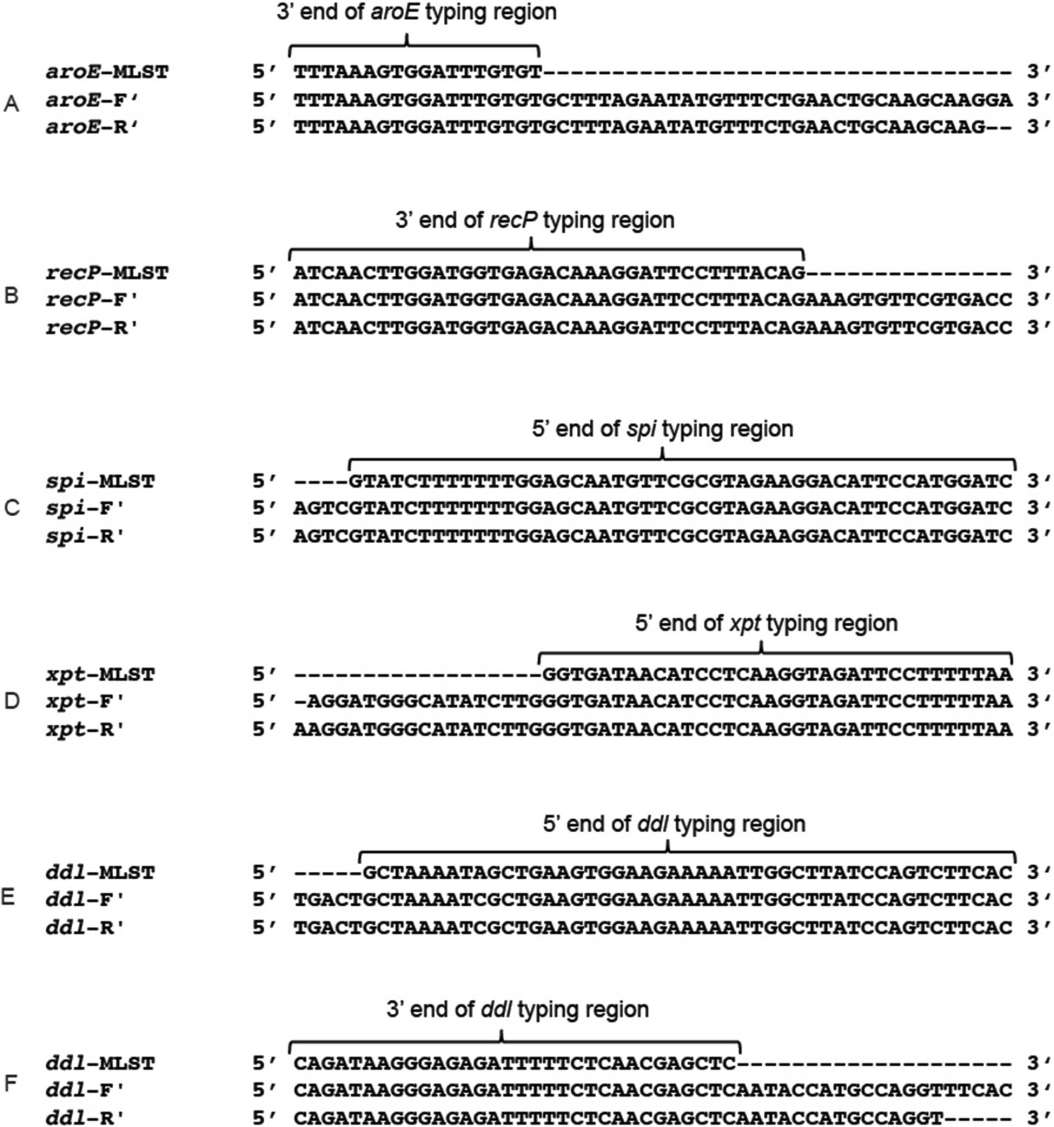
**5’ or 3’ end of the *S. pneumoniae *MLST typing region that is not obtained by both the forward and reverse standard primers aligned with the sequencing results from both the forward and reverse alternative primers.** Panels **(A)** through **(F)** depict the sequencing results of the alternative primers in relation to their corresponding typing region. The angle bracket and top sequence identify the 5’ or 3’ end of the typing region, the middle sequence is the result from sequencing with the forward alternative primer, and the bottom sequence is the result from sequencing with the reverse alternative primer.

A partial set of modified MLST primers for *S. pneumoniae* were designed and introduced by the US Centers for Disease Control (CDC) [12]. The CDC primers for *aroE*, the reverse primer for *recP*, and the forward primer of *ddl* each annealed within the coding sequence for the gene possessing the typing region, and were able to completely cover the required sequence. However, the CDC forward primer for *recP*, and both sets of *spi* and *xpt* primers annealed to regions of genomic DNA outside of their target gene. While these primers successfully sequenced the correct region, the highly plastic nature of pneumococcal genome suggests these genes may not always be in the same region, and in this case primers that bind outside of the gene may not always be specific to the target region of the genome [19]. To address sequencing errors potentially resulting from this phenomenon, the *recP* CDC forward primer was replaced with the standard MLST *recP* forward primer, as this primer annealed within the *recP* gene and can correctly sequence the typing region. Novel primers that annealed within the gene were also designed to replace the *spi* and *xpt* CDC primers. Lastly, the CDC reverse primer for *ddl* bound only 19 base pairs away from the typing region, and a modified primer binding 57 base pairs from the typing region was designed as a replacement. Analysis of the alternate primer sets (Table 2) using the same five test isolates revealed that, each primer set that was sufficiently down/upstream from the typing region was able to correctly amplify and sequence the appropriate DNA fragment (Figure 2). The effectiveness of the alternative primer set was subsequently validated through sequence typing of 105 diverse isolates collected by the Canadian Immunization Monitoring Program ACTive (IMPACT) surveillance network (Additional file 1: Table S1). In all cases investigated in this study, the modified primers were able to obtain the complete typing sequence, in both directions, for the gene/primer combinations not obtained by the standard primers.

**Table 2:** **Alternate primers used for amplifying and sequencing each of the seven genes for multi locus sequencing typing *S. pneumoniae***

**Typing gene**	**Primer sequences**	**Annealing temperature °C**
^1^*aroE* shikimate dehydrogenase	F: 5′-TCCTATTAAGCATTCTATTTCTCCCTTC	55
	R: 5′-ACAGGAGAGGATTGGCCATCCATGCCCACACTG	
^2^*gdh* glucose-6-phosphate dehydrogenase	F: 5′-ATGGACAAACCAGC(G/A/T/C)AG(C/T)TT	55
	R: 5′-GCTTGAGGTCCCAT(G/A)CT(G/A/T/C)CC	
^2^*gki* glucose kinase	F: 5′-GGCATTGGAATGGGATCACC	60
	R: 5′-TCTCCCGCAGCTGACAC	
^1,2^*recP* transketolase	F: 5′-GCCAACTCAGGTCATCCAGG	65
	R: 5′-TGCTGTTTCGATAGCAGCATGGATGGCTTCC	
^3^*spi* signal peptidase I	F: 5′-GAATTCATTTAAAAATTTCTTAAAAGAGTGG	50
	R: 5′-TTAAAATGTTCCGATACGGGTGATTGG	
^1^*xpt* xanthine phosphoribosyltransferase	F: 5′-TTAACTTTTAGACTTTAGGAGGTCTTATG	55
	R: 5′-CGGCTGCTTGCGAGTGTTTTTCTTGAG	
^1,3^*ddl* D-alanine-D-alanine ligase	F: 5′-TAAAATCACGACTAAGCGTGTTCTGG	65
	R: 5′-CGCTCGATTAGTTTTGGGTAGCTGATCCC	

^1^The *aroE* primers, the *recP* reverse primer, the *xpt* primers and the *ddl* forward primer were designed by the US Centers for Disease Control [12].

^2^The *recP* forward primer, and the *gdh* and *gki* primers are the originals developed by Spratt and Enright [11].

^3^The *spi* primers and the *ddl* reverse primer were designed as part of this study.

## Discussion

These results demonstrate that the current inability of the standard sequencing primers to effectively sequence the *S. pneumoniae* MLST typing regions is a result of how close the primers anneal to the typing region of the gene. When sequencing by Sanger chain termination capillary separation is employed, the base pairs immediately after the sequencing primer will not be clearly sequenced [20]. This is a characteristic of the size separating technology used by chain termination sequencing. When the terminated segments are separated based on size, there is poor resolution between the smaller fragments at the start of the sequence. This results in unclear and ambiguous sequencing results for approximately the first 20 – 50 base pairs of the sequence.

Next generation sequencing approaches such as 454, Illumina, and ABI function by determining the sequence for overlapping segments of 35 to 200 base pairs, depending on the specific method, and then assembling these segments into the complete sequence [21]. These next generation techniques have recently been applied to MLST with some success, however, the assembly process can be hindered by highly repetitive sequence in the overlapping sections of the sequence reads. This can potentially result in inaccurate assemblies within sequence typing regions. Additionally, the infrastructure and expertise required to employ next generation sequencing technologies still limits their accessibility to many research groups [21,22]. Given these limitations, and noting the number of recent studies still making unaltered reference to the standard primers, it remains valuable for researchers in this field to be more aware of the limitations presented by the standard MLST sequencing primers.

## Conclusion

The alternative primer set described here addresses the limitation of the standard *S. pneumoniae* MLST primers by annealing sufficiently far from the target region such that the sequence for the correct segment is consistently obtained. Clear documentation defining the limitations of the standard *S. pneumoniae* MLST primers and describing an effective set of alternative primers is of particular importance as automated Sanger capillary sequencing remains a highly optimized, and still widely employed method for *S. pneumoniae* MLST based studies.

## Methods

### *Streptococcus pneumoniae* strains and genomic preparation

Evaluation of the standard and alternative MLST primers was carried out on five randomly selected isolates from strains collected provided by the Canadian Immunization Monitoring Program ACTive (IMPACT) [23-26]. Isolates were obtained from patients aged 0 – 16 presenting with invasive pneumococcal infection at tertiary care centres across Canada; diagnosis was confirmed by positive *S. pneumoniae* culture from normally sterile body fluid (blood/cerebrospinal fluid). The IMPACT surveillance study has research ethics board approval at each participating centre to obtain demographic, clinical and microbiologic information on all cases without the requirement for written informed consent. *S. pneumoniae* strains were verified and serotyped as part of IMPACT’s routine surveillance protocol. The investigation described here was undertaken using IMPACT's 19A invasive strains, collected with ethical approval between 1991 and 2009. Strains were grown overnight at 5% CO_2_ on Columbia Blood Agar (prepared according to manufacturer’s instructions, Becton Dickinson and Company, Difco™, Sparks, Maryland, USA) plates with Optochin Disk (used according to manufacturer’s instructions, Sigma-Aldrich, Oakville, Ontario, Canada) susceptibility and the presence alpha hemolysis used for species verification. Genomic DNA was then isolated with the QIAamp DNA Mini Kit (used according to manufacturer instructions, Qiagen, Toronto, Ontario, Canada).

### Sequencing methodology

Each of the seven typing alleles was evaluated with both the standard (Table 1) and alternative (Table 2) MLST primers. PCR solutions were prepared for each primer set consisting of: 11 μl sterile distilled water, 2.5 μl of 10× reaction buffer (5 ml 1 M KCL, 5 ml 1 M (NH_4_)_2_SO_4_, 5 ml 2 M Tris–HCl pH 8.8, 5 ml 200 mM MgSO_4_, 5 ml 10% Triton X-100, water to 50 ml), 2.5 μl of 2 mM dNTPs, 2.5 μl of each primer at 5 μM, 1 unit pfu enzyme (Thermo Scientific, Ottawa, Ontario, Canada) and 2 μl of genomic DNA template at 50 – 300 ng/μl. All PCRs were performed in a BioRad (Mississauga, Ontario, Canada) Thermocycler with annealing temperatures specific to each primer set (Table 1 and 2). Amplification was verified by visualizing gene products with gel electrophoresis on a 1% ethidium bromide agarose gel with a voltage of 110 V for 25 minutes. Verified PCR products were purified with the E.Z.N.A Cycle Pure Kit (used according to manufacturer’s instructions OMEGA Biotek, Norcross, Georgia, USA). Purified products were subsequently verified via spectrophotometry (used according to manufacturer’s instructions NanoDrop 1000 Spectrophotometer, Thermo Scientific, Ottawa, Ontario, Canada). Purified samples with a concentration of greater than 3 ng/μl, and 260 nm/280 nm absorbance values between 1.0 and 2.0 were accepted to send for sequencing. Sequencing was carried out at both Macrogen Corporation, Rockville USA, and the University of Calgary, Calgary Canada, DNA Core Services facility.

### Assessing sequence coverage

The sequencing results were manually inspected for quality with the open source program 4Peaks, and sequence coverage was inspected by using the Multiple Sequence Alignment by Fast Fourier Transform (MAFFT) program, available through the European Bioinformatics Server [27]. MAFFT was used to align the forward and reverse sequence reads from each test primer set, and isolate, along with 5 known typing regions from the MLST database. The annealing site of each primer was identified by BLASTing the primer’s sequence against publically accessible *S. pneumoniae* genomic sequences available through the National Center for Biotechnology Information [28,29]. These results identified where each primer annealed relative to the typing region, and whether the sequencing resulting from the primer was able to consistently cover the required region. This full process was replicated twice for each primer set and each test isolate to confirm the reproducibility of the observations.

## Competing interests

The authors declare no competing financial or personal interests with respect to the presentation of these results.

## Authors’ contributions

PA contributed to the study’s conception, conducted the experiments, drafted the manuscript, and approved the final submission. Dr. OV is the IMPACT site co-investigator in Calgary Alberta, and was involved with the conception and design of the study, as well as the acquisition of the data. He also revised and approved the submitted manuscript. Dr. JK was involved in the conception and design of the study, and assisted in data acquisition. Dr. K also revised and approved the submitted manuscript. Dr. AS participated in the development of the project, provided technical support, and assisted in the acquisition of data and analysis of results. He revised and approved the submitted manuscript. Dr. JB is the IMPACT epidemiologist; she was involved in the conception and design of the study, provided the data and supervised the data analysis. She revised and approved the submitted manuscript. Dr. JA contributed substantially to the conception, implementation, and interpretation of the results presented in this study. Dr. JA, also revised and approved the submitted manuscript. All authors read and approved the final manuscript.

## Supplementary Material

Additional file 1: Table S1 *S. pneumoniae* strains sequence typed with alternative MLST primers. Click here for file
