# Supplementary material for: Effectiveness of the standard and an alternative set of Streptococcus pneumoniae multi locus sequence typing primers
Source: BMC Microbiol. 2014 Jun 3;14:143. doi: 10.1186/1471-2180-14-143 (PMC4057806; doi:10.1186/1471-2180-14-143)
Supplement: Additional file 1: Table S1 — S. pneumoniae strains sequence typed with alternative MLST primers. [file 1471-2180-14-143-S1.doc]

Additional file 1: Table S1 *S. pneumoniae* strains sequence typed with alternative MLST primers

| **^1^Strain No.** | **Year** | **Sequence Type** | **Province** |
| --- | --- | --- | --- |
| 1 | 1991 | 416 | Manitoba |
| 2 | 1991 | 9007 | Manitoba |
| 3 | 1991 | 416 | Manitoba |
| 4 | 1992 | 2220 | Manitoba |
| 5 | 1992 | 62 | Manitoba |
| 6 | 1992 | 9008 | Alberta |
| 7 | 1993 | 180 | Ontario |
| 8 | 1993 | 7229 | Manitoba |
| 9 | 1993 | 667 | Manitoba |
| 10 | 1993 | 199 | Quebec |
| 11 | 1993 | 876 | Quebec |
| 12 | 1994 | 9009 | Nova Scotia |
| 13 | 1994 | 9001 | Ontario |
| 14 | 1994 | 9010 | Ontario |
| 15 | 1994 | 2732 | Quebec |
| 16 | 1994 | 9002 | Quebec |
| 17 | 1994 | 490 | Quebec |
| 18 | 1995 | 58 | Alberta |
| 19 | 1995 | 416 | Quebec |
| 20 | 1995 | 415 | Ontario |
| 21 | 1995 | 199 | Alberta |
| 22 | 1996 | 199 | British Columbia |
| 23 | 1996 | 416 | Quebec |
| 24 | 1996 | 2013 | Ontario |
| 25 | 1996 | 416 | Ontario |
| 26 | 1997 | 2013 | Ontario |
| 27 | 1997 | 9011 | Nova Scotia |
| 28 | 1997 | 416 | Manitoba |
| 29 | 1997 | 9004 | Ontario |
| 30 | 1997 | 5282 | Quebec |
| 31 | 1998 | 876 | Quebec |
| 32 | 1998 | 58 | Manitoba |
| 33 | 1998 | 199 | Quebec |
| 34 | 1998 | 1848 | Quebec |
| 35 | 1998 | 416 | Ontario |
| 36 | 1999 | 9005 | Quebec |
| 37 | 1999 | 2464 | Ontario |
| 38 | 2004 | 667 | Ontario |
| 39 | 2003 | 276 | Quebec |
| 40 | 2003 | 276 | Quebec |
| 41 | 2005 | 2013 | British Columbia |
| 42 | 2005 | 9015 | Quebec |
| 43 | 2005 | 416 | Manitoba |
| 44 | 2006 | 416 | Alberta |
| 45 | 2006 | 63 | Quebec |
| 46 | 2006 | 2270 | Ontario |
| 47 | 2006 | 276 | Manitoba |
| 48 | 2006 | 63 | Quebec |
| 49 | 2006 | 2013 | Quebec |
| 50 | 2006 | 199 | Quebec |
| 51 | 2006 | 994 | Quebec |
| 52 | 2006 | 320 | Quebec |
| 53 | 2006 | 416 | Quebec |
| 54 | 2006 | 1201 | Ontario |
| 55 | 2006 | 1201 | Ontario |
| 56 | 2006 | 2343 | Ontario |
| 57 | 2006 | 994 | Quebec |
| 58 | 2006 | 276 | Quebec |
| 59 | 2007 | 9006 | Alberta |
| 60 | 2007 | 320 | Alberta |
| 61 | 2007 | 9012 | Alberta |
| 62 | 2007 | 276 | British Columbia |
| 63 | 2007 | 2013 | Quebec |
| 64 | 2007 | 63 | Quebec |
| 65 | 2007 | 320 | Quebec |
| 66 | 2007 | 416 | Quebec |
| 67 | 2007 | 63 | Quebec |
| 68 | 2007 | 416 | Manitoba |
| 69 | 2007 | 276 | Quebec |
| 70 | 2007 | 199 | Ontario |
| 71 | 2007 | 320 | Ontario |
| 72 | 2007 | 9013 | Saskatchewan |
| 73 | 2007 | 2013 | Alberta |
| 74 | 2007 | 2343 | Ontario |
| 75 | 2007 | 1201 | Ontario |
| 76 | 2007 | 2343 | Ontario |
| 77 | 2007 | 1201 | Ontario |
| 78 | 2007 | 200 | Ontario |
| 79 | 2007 | 416 | Quebec |
| 80 | 2007 | 172 | Quebec |
| 81 | 2008 | 320 | Alberta |
| 82 | 2008 | 276 | Quebec |
| 83 | 2008 | 2270 | Ontario |
| 84 | 2008 | 667 | Alberta |
| 85 | 2008 | 320 | Alberta |
| 86 | 2008 | 416 | Quebec |
| 87 | 2008 | 320 | Nova Scotia |
| 88 | 2008 | 1621 | British Columbia |
| 89 | 2008 | 320 | Alberta |
| 90 | 2008 | 276 | Quebec |
| 91 | 2008 | 276 | Quebec |
| 92 | 2008 | 193 | Quebec |
| 93 | 2008 | 2927 | Quebec |
| 94 | 2008 | 276 | Quebec |
| 95 | 2008 | 274 | Quebec |
| 96 | 2008 | 667 | Ontario |
| 97 | 2008 | 2345 | Ontario |
| 98 | 2008 | 2343 | Ontario |
| 99 | 2008 | 9003 | Ontario |
| 100 | 2008 | 4641 | Manitoba |
| 101 | 2008 | 9014 | Quebec |
| 102 | 2008 | 276 | Quebec |
| 103 | 2008 | 416 | Quebec |
| 104 | 2008 | 276 | Quebec |
| 105 | 2009 | 2472 | Ontario |

*^1^ Strain ID numbers only identify strains within the context of this study*

** sequence types > 9000 identify novel sequence types from this study*
